# *Open Biology* Editorial: What do you really think?

**DOI:** 10.1098/rsob.200334

**Published:** 2020-11-18

**Authors:** Jon Pines

If you will indulge me for a minute or two, I will just reach for my rose-tinted glasses. My views on what makes a good paper (https://blogs.royalsociety.org/publishing/incoming-editor-in-chief-for-open-biology-jon-pines-explains-how-to-make-a-good-paper-into-a-great-paper/) were formed by exemplars of clarity of thinking and clear logical argument, but what often impressed and—as important—excited me was a Discussion in which the authors put their conclusions in a wider context. Often, this involved broader speculation, always grounded in logic and based on sound data, but going one step further to explain how the study advanced our understanding of a fundamental concept, or how the study might be related to another area of science. Regrettably, page limits have tended to discourage this type of writing and some of the natural excitement of research inevitably lost. But now at *Open Biology*, no article limits mean that the main constraint will simply be the breadth of your ideas!

We are introducing a new section at the end of the Discussion in a section called ‘Opening Up’ where authors are actively encouraged to place their results in a wider context and discuss their broader implications. We particularly encourage discussion of how your findings may change existing pre-conceptions, or open up new ways to look at a problem. We want to transform the conservative trend where setting out how your results have altered your thinking is discouraged as ‘simply speculation’, to one where logical and grounded speculation is most welcome. We want your opinions!

This section will be only lightly peer-reviewed: it will be sense-checked but cannot be used to request further experiments. In and of itself, this will be an experiment, but we hope that *Open Biology* authors will take the opportunity to express both their insight and their excitement!

